# In-stream decomposition rates of Japanese knotweed ( *Reynoutria japonica* ) and jewelweed ( *Impatiens capensis* ) vary by inclusion or exclusion of macroinvertebrate shredders

**DOI:** 10.17912/micropub.biology.002158

**Published:** 2026-06-22

**Authors:** Anne Llewellyn, Matthew Wilson

**Affiliations:** 1 Ecology Program, Susquehanna University; 2 Freshwater Research Institute, Susquehanna University, Selinsgrove, PA, US

## Abstract

Leaf litter decomposition rates vary widely in stream ecosystems and understanding how decay over time ( *k* ) varies by species can be critical to estimating allochthonous inputs and temporal changes in energy availability in streams. We deployed leaf packs of Japanese knotweed ( *Reynoutria japonica* ) and jewelweed ( *Impatiens capensis* ) with fine (0.5 mm) and coarse (3.35 mm) mesh to exclude or include macroinvertebrate shredders, respectively, over an 8-week period. We found jewelweed decomposed 3-7 times faster than knotweed and jewelweed in coarse packs decomposed faster than any published stream litter rate. These results highlight the importance of understanding context-dependent herbaceous species decomposition.

**Figure 1. Decomposition curves for jewelweed and knotweed:** Figure: Decomposition rates and equation fit for (A) jewelweed excluding macroinvertebrates, (B) Jewelweed including macroinvertebrates, (C) Japanese knotweed excluding macroinvertebrates, and (D) Japanese knotweed including macroinvertebrates.

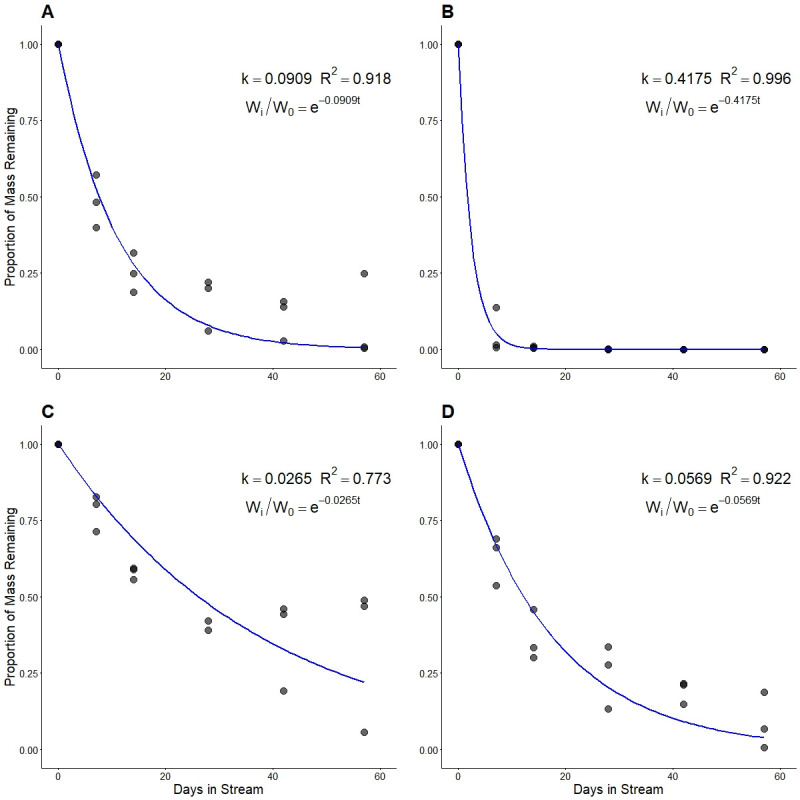



## Description

Leaf litterfall from riparian corridors is often the primary source of organic matter inputs into temperate headwater streams and critical to ecosystem function and food webs (Wallace et al., 2015). However, leaf litter decomposition rates vary widely in stream ecosystems, largely based on leaf quality (Leroy and Marks 2006), water temperature (Martínez et al., 2014), and biological processing (e.g., microbial and macroinvertebrate consumption; Webster and Benfield 1986). These processes and environmental conditions in conjunction influence the standing stocks of particulate organic matter in streams and the annual variability in allochthonous energy availability to food webs (e.g., Hare et al, 2024).

While there has been substantial research into processing of tree litterfall and how these influence stream functioning (Webster et al., 1995), there has been comparatively little research of herbaceous species. In addition, the influence of invasive species introductions, such as Japanese knotweed ( *Reynoutria japonica* ), are gaining attention for changing quality, quantity, and timing of inputs with the potential for cascading ecosystem effects (Robertson and Coll 2019; Cybill et al., 2020). However, because of the site-specificity in decomposition rates, it is difficult to generalize how replacement of native herbaceous species by invasives might alter decomposition rates without direct comparisons.

Our goal was to determine the difference in decomposition rates ( *k* , day ^-1^ ) between jewelweed ( *Impatiens capensis* ), an eastern North American native species, and *R. japonica* , a common riparian invasive of the same habitats. In addition, we compared these rates with inclusion or exclusion of macroinvertebrate shredders to separate the relative contributions of microbial and macroinvertebrate processing to *k* . We placed five, 5-gram leaf packs of each species with fine (0.5 mm) and coarse (3.35 mm) mesh in three riffles of a spring-fed headwater stream to Chillisquaque Creek in central Pennsylvania, USA. We collected one leaf pack from each treatment group and each riffle at one, two, four, six, and eight weeks post-placement in-stream on 7 October 2025 (n = 60). Post-collection, we rinsed leaves, oven-dried at 70ºC for 48 hours, ashed leave material at 550ºC for 60 minutes to measure ash free dry mass (AFDM), and calculated *k* based on proportion of AFDM remaining from each pack. We calculated decay curves and plotted results with base R and the ggplot2 package (R Core Team 2024; Wickham 2016). To test for significant differences in decay rates (as proportion mass remaining) between groups we used an analysis of covariance (ANCOVA) with collection date as a covariate and mesh size and leaf species as explanatory factors (i.e., leaf proportion remaining ~ mesh + species + collection date).

Overall, *I. capensis* in coarse leaf packs (allowing macroinvertebrate shredding) had dramatically faster decomposition than all other treatment groups, followed by fine mesh (excluding macroinvertebrates) for *I. capensis* , coarse mesh *R. japonica* , then fine mesh *R. japonica* (Figure 1). All groups were significantly different from each other with F _1,67_ = 21.42 and p << 0.001 for species; and F _1,67_ = 10.17 and p = 0.002 for mesh size when including date as a covariate (overall model adjusted R ^2^ = 0.62, F _3,67_ = 39.08, and p << 0.001). While these decomposition rates are higher than typically reported in the literature, our stream temperature was warmer and cumulative degree day higher than the only study we have identified comparing *R. japonica* decomposition to another *Impatiens* species ( *I. glandulifera* ) which found a similar ratio between these species (5.1x faster for *Impatiens* vs 3.4-7.3x faster in our study), yet overall decomposition rates were an order of magnitude slower (Kuglerová et al., 2017). By comparison, weekly mean water temperatures in our experiment ranged between 5.22-14.21ºC while Kuglerová et al. (2017) had water temperatures considerably lower, between 1.5-10.6ºC. Patterns were also consistent between mesh sizes within species, as the inclusion of macroinvertebrate shredders with coarse leaf packs was expected to increase litter decomposition rates (Webster and Benfield 1986).

These results underscore the importance of local variability in decomposition rates while filling a knowledge gap to allow for comparison by ratio across studies and species (e.g. Kennedy and El‐Sabaawi 2017). This also highlights the importance of understanding herbaceous species decomposition and expanding study species in more open canopy systems.

## Methods

Leaf collection and preparation

We collected pre-senescent (green) leaves from both species at the Center of Environmental Education and Research (CEER) at Susquehanna University in Selinsgrove, Pennsylvania. The leaves were cut from stalks at the petiole and air-dried in a climate-controlled space for two weeks, until remeasure of dry mass did not change over time. We filled leaf packs, both fine mesh (0.5 mm) packs, which allowed for exclusion of macroinvertebrates, and coarse mesh (3.35 mm) packs, which allowed for inclusion of macroinvertebrates, with approximately 5 g of dried leaves (5.0005 - 5.0859 g) for deployment.

Study site

We deployed leaf packs in a spring-fed unnamed headwater stream to Chillisquaque Creek at the Bucknell University Natural Area (BUNA) in Milton, Pennsylvania, USA. The three riffles were 30 ± 5 m apart and the streambed was primary composed of gravel and cobble. Leaf pack chains consisted of five packs for each treatment type placed in random order and location within each riffle to avoid potential for spatial bias. Packs were fully submerged underwater to ensure no movement and separated fully from each other to ensure no overlapping of individual packs. Packs were collected 1, 2, 4, 6, and 8 weeks after original deployment on October 7 ^th^ , 2025.

Sample processing

We washed leaf material in a 350 µm sieve to remove sediment and macroinvertebrates, then dried leaves at 70°C for two days. After recording dry weight, samples were homogenized in a Wiley Mini-Mill, with three 0.25 g subsamples of each pack (or maximum possible number of 0.25 g subsamples from remaining mass) ashed at 550°C for 1 hour, and mean organic content was used to calculate remaining AFDM. We compared this to initial AFDM for each leaf species to calculate proportion remaining by pack and collection date.
